# Differences in leaf heat and drought tolerance but not cold tolerance between karst and non-karst forest plants

**DOI:** 10.1016/j.pld.2025.08.006

**Published:** 2025-08-26

**Authors:** Qiufeng Ning, Yin Wen, Hui Liu, Jiawei Li, Yunpeng Nie

**Affiliations:** a Guangxi Key Laboratory of Forest Ecology and Conservation, State Key Laboratory for Conservation and Utilization of Subtropical Agro-bioresources, College of Forestry, Guangxi University, Nanning 53004, China; b Institute of Subtropical Agriculture, Chinese Academy of Sciences, Changsha 410125, China; c Guangxi Key Laboratory of Karst Ecological Processes and Services, Huanjiang Observation and Research Station for Karst Ecosystem, Chinese Academy of Sciences, Hechi 547100, China; d Guangdong Provincial Key Laboratory of Applied Botany, State Key Laboratory of Plant Diversity and Specialty Crops, Key Laboratory of National Forestry and Grassland Administration on Plant Conservation and Utilization in Southern China, South China Botanical Garden, Chinese Academy of Sciences, Guangzhou, 510650, China

**Keywords:** Photosynthetic heat tolerance, Photosynthetic cold tolerance, Turgor loss point, Heat stress, Leaf traits, Karst forests

## Abstract

Ongoing climate change and increasingly frequent extreme precipitation events pose greater threats to plant survival. Plants in the karst environment may face heightened risks because shallow soils and poor water retention amplify drought and temperature stresses, yet their physiological tolerances remain poorly understood. In this study, we aimed to investigate the leaf physiological tolerance strategies to drought and temperature stress in plants from karst versus non-karst forests, and to quantify the relative contributions of lithology and phylogeny to variation in these tolerances. In this study, we measured leaf photosynthetic heat and cold tolerance, leaf turgor loss points, and morphological and anatomical traits in 39 dominant woody species from karst and non-karst forests in Guangxi, China. We used Welch’s t-tests to compare leaf trait differences between forest types, evaluated the trait relationships with Pearson correlation analysis, and partitioned the contributions of lithology and phylogeny to trait variation using phylogenetic eigenvector regression (PVR). Karst species exhibited more negative leaf turgor loss points (π_tlp_) and lower heat tolerance (T50_heat_) than non-karst species, whereas cold tolerance (T50_cold_) did not differ between habitats. Leaf thickness (LT) and leaf mass per area (LMA) were positively correlated with T50_heat_, suggesting that higher structural investments enhance heat tolerance, but are not correlated with T50_cold_ and π_tlp_. Phylogeny predominantly explains the variation in T50_cold_ and the second principal component (PC2), whereas lithology primarily drove variation in π_tlp_ and T50_heat_. Because karst species have lower heat tolerance, they may face a higher risk of thermal damage under future climate warming.

## Highlights

• Measured leaf heat, cold and drought tolerances in 39 woody species from karst and non-karst forests in Guangxi, China.
• Karst species exhibited stronger drought tolerance but lower heat tolerance compared to non-karst species.
• Leaf thickness and mass per area positively correlated with heat tolerance, but not with cold or drought tolerance.
• Cold tolerance was primarily shaped by phylogeny, while drought and heat tolerance were mainly influenced by lithology.
• Karst plants may be more vulnerable to thermal stress under future climate warming.

## 1 Introduction

Temperature and water are critical environmental factors that directly influence plant growth and distribution (Nievola et al., 2017; Wen et al., 2018; Jiao et al., 2021). In the context of global climate change, the rising frequency of extreme temperature and drought events poses significant threats to plant survival and has been contributing to widespread forest decline and tree mortality (Allen et al., 2010; Arnold et al., 2021; Ayala-Jacobo et al., 2021; Marchin et al., 2022). The latest IPCC report indicates that, if current greenhouse gas emission trends persist, the global average surface temperature is projected to increase by 1.5–2 °C by the end of this century (Kikstra et al., 2022). This rise is expected to result in a higher frequency and intensity of extreme temperature and drought events, which will, in turn, exert severe impacts on ecosystems (Zandalinas et al., 2021; Wen et al., 2023; Sato et al., 2024). Therefore, elucidating the mechanisms of plant adaptation to coupled temperature and water stress is vital for predicting their responses to climate change, modeling distributional shifts, and developing effective ecological conservation strategies.

Plants have evolved diverse physiological tolerances, including heat, cold, and drought tolerance, to cope with multiple and often simultaneous environmental stresses (Niinemets and Valladares, 2006; Grossman, 2023). However, current research predominantly focuses on the response mechanisms of single tolerance traits, with only approximately 5% of studies simultaneously addressing heat and cold tolerance, and about 6% examining relationships between heat tolerance and water availability (Geange et al., 2021). Furthermore, most of these studies have been conducted in temperate or tropical ecosystems, while extreme habitats characterized by excessive cold, drought, heat, or unique geological features—such as karst ecosystems—remain largely understudied. As a result, our understanding of plant tolerance under multiple, interacting environmental stresses remains limited. Previous studies have shown that different tolerances can interact and evolve synergistically, e.g., plants in cold regions tend to exhibit enhanced cold and drought tolerance, while species in arid environments are more heat tolerant (Bansal et al., 2016; Münchinger et al., 2023). Nonetheless, trade-offs between tolerances may also occur, limiting plant performance under multiple simultaneous stresses and constraining the ability of plants to optimize tolerance across different environmental challenges (Stahl et al., 2013; Puglielli et al., 2023). Therefore, the relationships among different physiological tolerances remain poorly understood.

Leaf morphological and anatomical traits play a pivotal role in plant adaptation to drought and temperature stress by regulating temperature and water exchange, thereby forming diverse ecological strategies (Stahl et al., 2014; Valliere et al., 2023). First, leaves can adjust the thickness of the boundary layer, thereby affecting the rate of heating or cooling, where larger leaf width (LW) and leaf area (LA) increase boundary layer thickness, reduce heat exchange with the air, and increase the risk of thermal damage (Jones, 2014; Slot et al., 2021). Second, leaves may reduce water loss and conserve water by increasing leaf thickness (LT) and decreasing LA, aiding survival in arid environments (Afzal et al., 2017; Maréchaux et al., 2020). Additionally, leaf mass per area (LMA) serves as a central trait of plant physiological tolerance to drought and temperature stress. Leaves with low LMA are typically thinner, losing water more rapidly in drought but facilitating rapid growth and photosynthesis in moist environments (De La Riva et al., 2016). While high LMA leaves are more compact with thicker cell walls, providing greater tolerance to extreme high temperatures and drought (De La Riva et al., 2016; Zhang et al., 2024b). These findings suggest that key leaf morphological and anatomical traits are crucial for plant adaptation to varying stress conditions, and more research is needed to reveal the underlying mechanisms.

The karst region in southwestern China is the largest and most typical contiguous karst area in the world (Cai, 1997, Lv et al., 2023), with the northern tropical karst forests of Guangxi being an important component. Plants in this region face unique environmental pressures, including exposed bedrock, shallow soils, and high concentrations of calcium (Ca) and magnesium (Mg), as well as frequent human disturbances (Wang et al., 2018; Tang et al., 2021). Critically, the extensive fissures and pores in karst landscapes result in severe water shortages (Wilcox et al., 2008, Yao et al., 2001; Guan et al., 2023), making vegetation highly vulnerable to temperature and water stress (Wang et al., 2023a). Consequently, global climate change is expected to further intensify these stresses, posing even greater survival challenges for the already vulnerable and sensitive karst ecosystems (Seddon et al., 2016; Zhang et al., 2022). To cope with such harsh environments, plants in karst regions are thought to have developed specific physiological tolerances. In addition, phylogeny plays an important role in shaping and constraining the evolution of physiological tolerance through phylogenetic conservatism. However, the extent to which lithology (karst environment) and phylogeny explain variation in the physiological tolerance of karst species remains unclear.

Comparing plant species from karst and adjacent non-karst forests under similar climatic conditions provides a robust approach to identifying the unique adaptations of karst species. In southwestern China, the close proximity of these contrasting habitats under comparable climates creates a natural laboratory to directly investigate how lithology shapes plant physiological adaptations. In this study, we focused on dominant woody species from two karst and non-karst forests in the northern tropical region of Guangxi. We measured leaf physiological tolerance to drought and temperature stress, along with key morphological and anatomical traits, to explore the mechanisms of plant adaptation under similar climatic but contrasting lithological conditions, thereby providing a scientific basis for the conservation and restoration of karst forests. Specifically, the study addresses two scientific questions: (1) How do leaf physiological tolerances to drought and temperature differ between karst and non-karst species, and how are these differences shaped by lithology and phylogeny? (2) How are leaf physiological tolerances associated to morphological and anatomical traits? We hypothesize that, due to the unique and harsh environmental pressures in karst habitats, karst species will exhibit greater leaf physiological tolerance to drought and heat than non-karst species. We further expect that these tolerances to be closely associated with key leaf morphological and anatomical traits, such as LT and LMA.

## 2 Materials and methods

### 2.1 Study sites

Experiments were conducted at two nature reserves in Guangxi, China: Nonggang (22.47°N, 106.95°E; elevation 273 m, karst) and Shiwandashan (21.84°N, 107.89°E; elevation 558 m, non-karst). These forest sites are geographically close and share similar climatic conditions, with Nonggang having a mean annual temperature of 21.8 °C and annual precipitation of 1666.2 mm, and Shiwandashan having a mean annual temperature of 19.3 °C and annual precipitation of 1946.6 mm (Table S1). However, the two reserves differ markedly in lithology. Nonggang is typical karst topography, characterized by shallow soils rich in Ca and Mg (Li et al., 2023), and their vegetation is tropical rainforest. In contrast, the Shiwandashan features non-karst topography, primarily consisting of sedimentary, metamorphic, and igneous rocks, and with a northern tropical semi-evergreen seasonal rainforest and moist rainforest.

### 2.2 Species selection and sampling

Based on community survey data, we selected the 19 and 20 most abundant evergreen woody species from karst and non-karst forests, respectively. Heat tolerance experiments and turgor loss points (π_tlp_) measurements were conducted from July to August 2023, while cold tolerance experiments were performed from January to February 2024, aligning with the corresponding seasonal stresses periods. Turgor loss point was measured in the same season as heat tolerance assessments because these two stressors typically co-occur.

For each species, we sampled three to five healthy, mature individuals. Leaf collection was conducted between 07:30 and 09:30 on sunny days to minimize the effects of diurnal variation in water content and photosystem activity. Approximately 60 fully expanded, sun-exposed, and mature leaves were collected per species, immediately placed in black plastic bags with damp paper towels, sprayed with water to maintain high humidity, and promptly transported to the laboratory for subsequent measurements. Due to specific experimental requirements and practical constraints, the number of leaves per treatment varied slightly across experiments (typically five to ten leaves). To ensure representative sampling, leaves for each treatment were consistently collected from three to five healthy individuals.

### 2.3 Photosynthetic heat tolerance

Following Krause et al. (2010), we assessed photosynthetic heat tolerance by measuring the temperature at which the maximum quantum yield of PSII (Fv/Fm) begins to decrease (Tcrit_heat_) and the temperature that causes a 50% decrease in Fv/Fm (T50_heat_) under high-temperature conditions (Kalaji et al., 2014; O'sullivan et al., 2017). Leaves were cut into 4 cm^2^ squares (or used whole if smaller) and dark-adapted for 1 h. Fv/Fm was then measured at room temperature (approximately 28 °C) using a LI-600 portable fluorometer (LI-COR, Lincoln, USA); only leaves with an initial Fv/Fm > 0.7 were used to exclude photoinhibition samples. Each leaf piece was subjected to only one temperature treatment to avoid cumulative heat damage. Leaf pieces were placed into separate sealed plastic bags (two non-overlapping pieces per bag) to ensure uniform heating, and exposed to water bath treatments at one of the following temperatures: 36, 40, 44, 46, 48, 50, 52, 54, 56, or 60 °C, for 15 min (Eight replicates per species at each temperature). After treatment, the samples were dark-adapted for 24 h before measuring the Fv/Fm. Tcrit_heat_ and T50_heat_ were calculated by fitting the Weibull model using the method and R code developed by Perez et al. (2021).

### 2.4 Photosynthetic cold tolerance

Following Sierra-Almeida et al. (2009), we assessed photosynthetic cold tolerance by measuring the temperature at which the maximum quantum yield of PSII (Fv/Fm) begins to decrease (Tcrit_cold_) and the temperature that causes a 50% decrease in Fv/Fm (T50_cold_) under low-temperature conditions (Knaupp et al., 2011; Pérez et al., 2014). Leaves were dark-adapted for 1 h and their Fv/Fm values were measured at room temperature conditions (approximately 18 °C) using a LI-600 portable fluorometer. Each leaf sample was subjected to only one cold-temperature treatment to prevent cumulative cold injury. Fully expanded, mature leaves were then attached to Type K thermocouples (supplied with the thermal data logger, Yongpeng, YP5064, China) and fixed in a 10 mm thick foam box. The box was then placed into a variable-frequency refrigerator (Aucma, BD-186WPHNXI, China) pre-cooled to one of the following temperatures: 4, 0, −2, −4, −6, −8, −12, −16, or −24 °C (Five replicates per species at each temperature). The foam box buffered heat exchange, allowing leaves to cool gradually and minimizing the effects of rapid temperature decline (Arnold et al., 2021). To account for internal temperature fluctuations, real-time leaf temperatures were monitored via thermocouples, and used for subsequent data fitting. Each temperature treatment lasted for 2 h, after which the foam box containing the leaves was removed and placed at 4 °C to allow gradual warming and minimize rapid freeze-thaw injury. The leaves were then dark-adapted at 4 °C for 24 h before final Fv/Fm measurement. Tcrit_cold_ and T50_cold_ were calculated using a Weibull model fitted with a modified version of the R code from Perez et al. (2021).

### 2.5 Turgor loss point

Drought tolerance was assessed using π_tlp_, following the protocol of Bartlett et al. (2012), a parameter validated as biologically meaningful in drought response analyses (Bartlett et al., 2014, 2016). Sampling was conducted following the same procedure as for heat tolerance experiments. Mature, well-hydrated leaves were collected in the early morning and rehydrated in the laboratory for approximately 20 min to ensure full turgor. For each leaf, small discs were punched from the central lamina (ten replicates per species), wrapped in aluminum foil, labeled, and immediately flash-frozen in liquid nitrogen. After 2 min, the samples were removed, punctured approximately 20 times with a fine needle, and promptly placed in the sample chamber of a osmometer (Wescor, Vapor 5600, USA) to measure the osmotic potential at full turgor (π_osm_). The π_tlp_ was calculated using the following formula: π_tlp_ = 0.832π_osm_ − 0.631 (Bartlett et al., 2012).

### 2.6 Leaf morphological and anatomical traits

For each species, we selected ten healthy and mature leaves for the measurement of morphological and anatomical traits. After carefully removing surface impurities, the leaves were scanned using a flatbed scanner (Epson, V850 Pro, Japan). Leaf width (LW, cm), leaf length (LL, cm), leaf perimeter (LP, cm), and leaf area (LA, cm^2^) were measured from the scanned images using ImageJ software (v.1.52n). The scanned leaves were then placed into pre-labeled envelopes and dried in an oven (Thermo Fisher Scientific, OMH400, USA) at 75 °C for 48 h before being weighed to obtain the leaf dry weight (LDW, g). Meanwhile, additional healthy and mature leaves were selected, from which the main vein was removed. Cross-sections were prepared using a double-edged stainless-steel blade to create temporary slides for observation under a light microscope. Leaf anatomical parameters—including leaf thickness (LT, μm), upper epidermis thickness (Tup, μm), lower epidermis thickness (Tlow, μm), palisade mesophyll thickness (Tp, μm), and spongy mesophyll thickness (Ts, μm)—were measured in micrometers using ImageJ software on temporary slides. Finally, we calculated the leaf length-to-width ratio (L/W), the palisade-to-spongy mesophyll thickness ratio (P/S), and the leaf mass per area (LMA, g/m^2^). All the traits measured are listed in Table 1.

**Table 1:** Abbreviations, descriptions, and units of all the traits measured in this study.

Abbreviation	Description	Unit
Tcrit_heat_	The high-temperature at which the maximum photochemical quantum yield of photosystem II (Fv/Fm) begins to decrease	°C
T50_heat_	The high-temperature that causes a 50% decrease in the maximum photochemical quantum yield of photosystem II (Fv/Fm)	°C
Tcrit_cold_	The low-temperature at which the maximum photochemical quantum yield of photosystem II (Fv/Fm) begins to decrease	°C
T50_cold_	The low-temperature that causes a 50% decrease in the maximum photochemical quantum yield of photosystem II (Fv/Fm)	°C
π_tlp_	Leaf water potential at turgor loss	MPa
Tup	Upper epidermis thickness	μm
Tlow	Lower epidermis thickness	μm
Tp	Palisade mesophyll thickness	μm
Ts	Spongy mesophyll thickness	μm
P/S	The palisade-to-spongy mesophyll thickness ratio	Dimensionless
LT	Leaf thickness	μm
LW	Leaf width	cm
LL	Leaf length	cm
L/W	Leaf length-to-width ratio	Dimensionless
LP	Leaf perimeter	cm
LA	Leaf area	cm²
LMA	Leaf mass per area	g/m²

### 2.7 Statistical analysis

All data analyses and visualizations were conducted in R (v.4.3.3; R Core Team, 2023). To explore the covariation of plant traits across the two habitats, principal component analysis (PCA) was applied to all measured traits, and the relationships among leaf physiological tolerance traits and morphological and anatomical traits were evaluated using Pearson correlation analysis. Differences in all measured traits between the two habitats were assessed using Welch's two-sample t-tests.

To examine phylogenetic signals and quantify the effects of phylogeny on trait variation, we constructed a phylogenetic tree using the V.PhyloMaker package (v.0.1.0). Phylogenetic signals for each trait were assessed by calculating Blomberg’s K values (Blomberg et al., 2003) using the picante package (v.1.8.2). To further partition the variance in physiological tolerance traits and principal components (PC1 and PC2), and to quantify the extent to which lithology and phylogeny explain trait variation, we applied phylogenetic eigenvector regression (PVR) (Desdevises et al., 2003; Liu et al., 2024). PVR fits a single linear regression model to analyze a dependent variable (trait y) with two predictors: lithology (karst or non-karst environments) and phylogenetic eigenvectors derived from a phylogenetic distance matrix. The variance in y was partitioned into four components: [a] + [b] + [c] + [d], where [a] is the variation explained by lithology, [b] is the shared variation explained by both lithology and phylogeny, [c] is the variation explained by phylogeny, and [d] is the unexplained variance.

## 3 Results

### 3.1 Differences in leaf traits between karst and non-karst plants

Karst species exhibited significantly lower photosynthetic heat tolerance (T50_heat_: 49.97 °C vs. 50.92 °C; Tcrit_heat_: 40.48 °C vs. 45.74 °C) and more negative turgor loss point (π_tlp_: −1.98 MPa vs. −1.40 MPa) compared to non-karst species. In contrast, no significant differences were observed in T50_cold_ and Tcrit_cold_ between the two habitats (Table 2).

**Table 2:** Results of mean and t-tests on traits between Karst and Non-karst species.

Traits	Karst (*n* = 19)	Non- karst (*n* = 20)	t	*p*
T50_heat_ (°C)	49.97 ± 0.38	50.92 ± 0.26	−2.06	< 0.05 ∗
Tcrit_heat_ (°C)	40.48 ± 0.71	45.74 ± 0.59	−5.68	< 0.01∗∗
T50_cold_ (°C)	−8.72 ± 0.60	−7.19 ± 0.50	−1.96	0.06
Tcrit_cold_ (°C)	0.18 ± 0.79	−1.83 ± 0.62	2.01	0.0523
π_tlp_ (MPa)	−1.98 ± 0.11	−1.40 ± 0.05	−4.73	< 0.01∗∗
Tup (μm)	21.37 ± 1.27	32.58 ± 2.72	−3.73	< 0.01∗∗
Tlow (μm)	16.66 ± 0.81	24.58 ± 2.09	−3.53	< 0.01∗∗
Tp (μm)	42.20 ± 3.48	55.00 ± 5.09	−2.07	< 0.05 ∗
Ts (μm)	81.59 ± 11.28	131.41 ± 15.07	−2.65	< 0.05 ∗
P/S	0.69 ± 0.11	0.48 ± 0.04	1.79	0.09
LT (μm)	165.05 ± 12.59	241.51 ± 17.63	−3.53	< 0.01∗∗
LW (cm)	5.54 ± 0.76	3.80 ± 0.21	2.21	< 0.05 ∗
LL (cm)	11.92 ± 1.03	10.73 ± 0.54	1.03	0.31
L/W	2.38 ± 0.14	2.90 ± 0.12	−2.84	< 0.01∗∗
LP (cm)	31.09 ± 3.53	24.77 ± 1.21	1.69	0.10
LA (cm^2^)	51.20 ± 11.77	27.14 ± 2.59	2.00	0.06
LMA (g/m^2^)	66.57 ± 5.78	82.64 ± 6.34	−1.87	0.07

Data are mean ± SE, sample sizes (n) are given in brackets. ∗ and ∗∗ indicates *p* < 0.05 and *p* < 0.01, respectively. Abbreviations are in Table 1.

For leaf morphological and anatomical traits, karst species had significantly lower values of LT, Tup, Tlow, Tp, Ts, and L/W, but greater LW. No significant differences were found in other traits between habitats (Table 2).

**Fig. 1:**
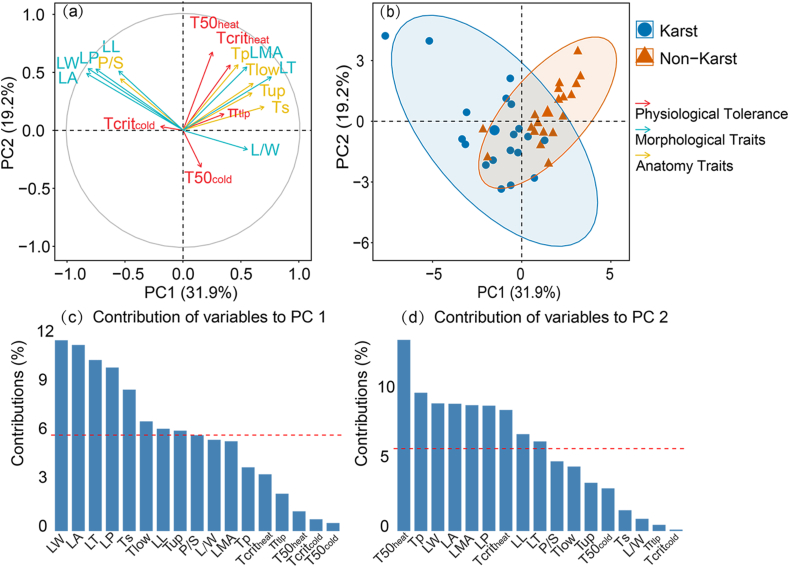
Principal component analysis of Karst and Non-karst evergreen woody species based on 17 leaf functional traits. (a) Loading of first two principal components (PC), and (b) scores for each species along PC1 and PC2, with Karst species (blue circles) and Non-karst species (orange triangles). Vectors in the (a) represent individual leaf functional traits, colored by categories: red for Physiological Tolerance, green for Morphological Traits, and yellow for Anatomy Traits. (c and d) Contribution of variables to the first and second PC, respectively ranked from high to low. Red dashed lines in (c) and (d) indicate average contribution of each variable. Abbreviations are in Table 1.

Principal component analysis (PCA) revealed clear separation between karst and non-karst species along the first two principal components. PC1 accounted for 31.9% of the total variance and was primarily influenced by morphological traits such as LW, LA, LT, and LP. PC2 explained 19.2% of the variance and was predominantly driven by photosynthetic heat tolerances (T50_heat_ and Tcrit_heat_) and Tp, LW, and LA. Together, PC1 and PC2 contributed 51.1% of the total variance (Fig. 1). Overall, the distinctions between karst and non-karst forests were mainly driven by heat tolerance and leaf morphological traits.

### 3.2 Drivers of leaf physiological tolerances traits and their associations with morphological and anatomical traits

**Fig. 2:**
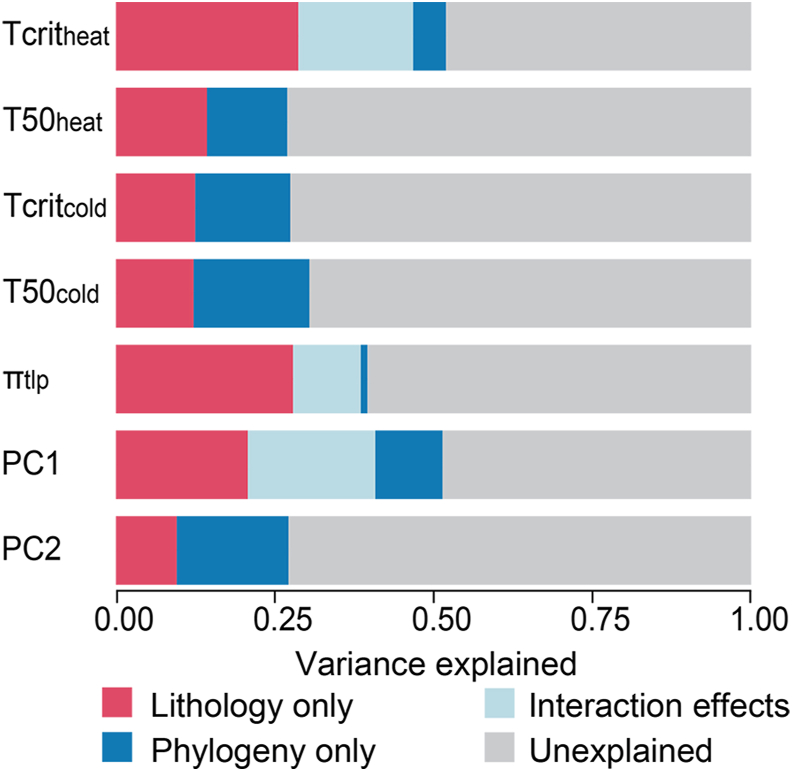
Relative contributions to total variance in plant functional traits of studied Karst and Non-karst species. Models are based on lithology variables, phylogeny factors. The bars represent the variance explained by pure lithology (red), pure phylogeny (blue), interaction effects from two factors (light blue), and unexplained variance (grey). Each row corresponds to a specific plant functional trait, and the segments within each bar indicate the proportion of variance attributed to each factor or interaction. Abbreviations are in Table 1, detailed parameters in Table S2.

Among physiological tolerances, π_tlp_ (K = 0.69, *p* = 0.007) and T50_cold_ (K = 0.52, *p* = 0.043) showed significant phylogenetic signals (Table 3). PVR analysis revealed that lithology explained a higher proportion of variance for π_tlp_ (both single and joint effects R^2^ = 0.386, hereafter as joint R^2^) and PC1 (joint R^2^ = 0.409), while phylogeny significantly explained the variance for T50_cold_ (joint R^2^ = 0.156) and PC2 (joint R^2^ = 0.140). Despite this, a substantial proportion of variation in all traits remained unexplained, ranging from 48.0% to 76.3%, with particularly high residuals for T50_heat_, Tcrit_cold_, and PC2 (Fig. 2; Table S2).

**Table 3:** Phylogenetic signal (Blomberg’s K) of the measured 17 leaf functional traits.

Traits	Blomberg’s K	*p*
T50_heat_ (°C)	0.24	0.61
Tcrit_heat_ (°C)	0.43	0.12
**T50_cold_ (°C)**	**0.52**	**0.043**
Tcrit_cold_ (°C)	0.45	0.09
**π_tlp_ (MPa)**	**0.69**	**0.007**
Tup (μm)	0.35	0.34
Tlow (μm)	0.19	0.82
Tp (μm)	0.23	0.66
Ts (μm)	0.53	0.06
P/S	0.38	0.28
LT (μm)	0.42	0.15
LW (cm)	0.45	0.26
LL (cm)	0.28	0.50
L/W	0.27	0.47
LP (cm)	0.32	0.46
LA (cm^2^)	0.40	0.38
LMA (g/m^2^)	0.27	0.53

Bold indicates significant phylogenetic signals (*p* < 0.05). The phylogenetic tree used for this analysis is presented in Figure S2.

**Fig. 3:**
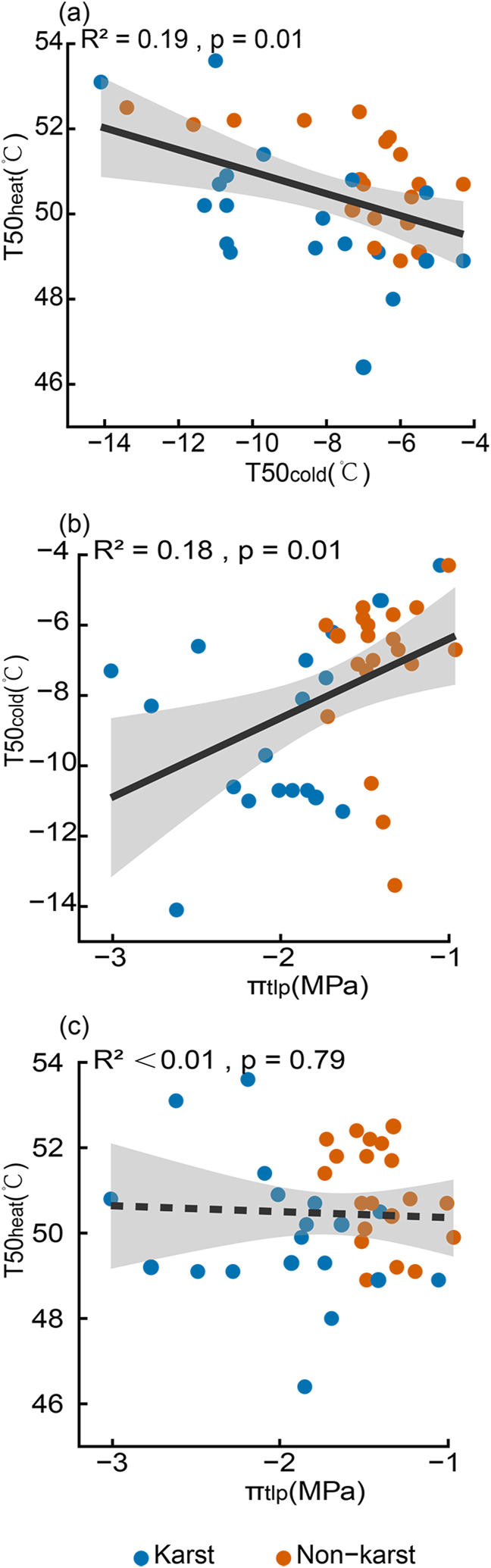
Correlations of (a) the high-temperature that causes 50% decrease of the maximum photochemical quantum yield of photosystem II (T50_heat_) and the low-temperature that causes 50% decrease of the maximum photochemical quantum yield of photosystem II (T50_cold_), (b) T50_cold_ and the leaf water potential at the turgor loss (π_tlp_), (c) T50_heat_ and π_tlp_ for the 39 evergreen woody species of Karst (blue) and Non-karst (orange). Significant correlations are shown as solid lines, and non-significant ones are shown as dashed lines.

**Fig. 4:**
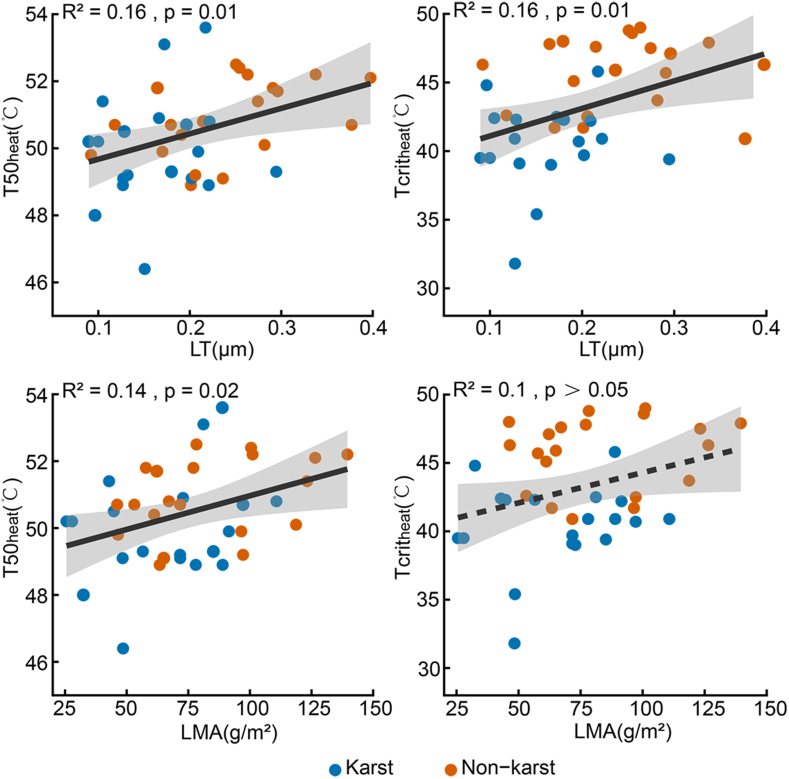
Correlations of the high-temperature at which the maximum photochemical quantum yield of photosystem II (Fv/Fm) begins to decrease (Tcrit_heat_) and high-temperature that causes 50% decrease of the maximum photochemical quantum yield of photosystem II (T50_heat_) with leaf thickness (LT) and Leaf mass per area (LMA) in 39 evergreen woody species of Karst (blue) and Non-karst (orange). Significant correlations are shown as solid lines, and non-significant ones are shown as dashed lines.

T50_cold_ was significantly negatively correlated with T50_heat_ (R^2^ = 0.19, *p* = 0.01) and significantly positively correlated with π_tlp_ (R^2^ = 0.18, *p* = 0.01), while T50_heat_ was not correlated with π_tlp_ (R^2^ < 0.01, *p* = 0.79) (Fig. 3). Among leaf morphological and anatomical traits, LT and LMA were both positively correlated with T50_heat_ (R^2^ = 0.16, *p* = 0.01; and R^2^ = 0.14, *p* = 0.02, respectively), while LT was also positively correlated with Tcrit_heat_ (R^2^ = 0.16, *p* = 0.01) (Fig. 4). Additionally, Tp was positively correlated with both T50_heat_ and Tcrit_heat_, while Tup and Tlow were both positively correlated with π_tlp_ (Fig. S1). No other significant correlations were observed between other morphological and anatomical traits and physiological tolerances.

## 4 Discussion

### 4.1 Divergent plant adaptation strategies in karst and non-karst regions

In line with our hypothesis, karst species exhibit more negative π_tlp_, consistent with previous findings that karst plants tend to maintain lower leaf water potential and π_tlp_ than non-karst species (Chen et al., 2015; Wan et al., 2023). This suggests that π_tlp_ is likely shaped by the unique selection pressures of karst habitats, where complex geological structures and widespread exposed bedrock create heterogeneous microhabitats with shallow soils and poor water retention (Fu et al., 2019; Qi et al., 2021). These conditions impose persistent drought stress, driving the evolution of specialized drought tolerance strategies. However, contrary to our expectations, karst species showed lower photosynthetic heat tolerance than non-karst species. This may be because, under long-term drought conditions, leaf photosynthesis is more constrained by water availability than by temperature (Chaves et al., 2002; Pinheiro and Chaves, 2011). Besides, the exposed rocky environment and intense solar radiation in karst habitats likely result in higher leaf temperatures in karst species compared to non-karst species. Consequently, both the elevated leaf temperatures and lower heat tolerance of karst plants act to narrow their thermal safety margin, making them more susceptible to thermal stress. This vulnerability is further exacerbated by their thinner leaves, which provide reduced thermal buffering capacity and lower resistance to extreme heat (Leigh et al., 2012; Marchin et al., 2022). Consequently, despite their enhanced drought tolerance, karst species may be more vulnerable to thermal damage under global warming scenarios. Cold tolerance did not differ significantly between the two habitats, likely reflecting long-term adaptation to similar regional climatic conditions, which impose comparable cold selective pressures. Cold tolerance is strongly influenced by evolutionary history and niche conservatism (Hawkins et al., 2014) and can be enhanced through cold acclimation (Cavender-Bares et al., 2005; Ayala-Jacobo et al., 2021). Thus, similar climatic conditions among our study sites appear to constrain divergence in cold tolerance and promote convergent cold tolerance strategies in both karst and non-karst species.

### 4.2 Lithology and phylogeny in explaining trait variation

The results for T50_cold_ and π_tlp_ show significant phylogenetic signals, supporting evolutionary conservatism in plant adaptations to cold and drought, consistent with results of Lancaster and Humphreys (2020) and Liu et al. (2024). Although π_tlp_ exhibits evolutionary conservatism, PVR analysis reveals that lithology—the unique geological conditions of karst habitats—accounts for 38.6% of its variance, indicating that strong environmental selection may drive the adaptive evolution of this trait. Similarly, karst lithology drives the evolution of traits such as deep root systems and high leaf hydraulic conductance, which enhance drought tolerance by accessing water in karst and maintaining efficient water transport fissures in dry soils (Du et al., 2023; Wan et al., 2023; Ma et al., 2024). In contrast, neither T50_heat_ nor the most leaf morphological and anatomical traits display significant phylogenetic signals. Instead, PVR analysis further indicates that T50_heat_ and PC1 (which primarily represents leaf morphology) are more strongly influenced by lithological selection rather than phylogenetic constraints. Several studies suggest that heat tolerance is mainly driven by environmental factors and is closely associated with leaf morphological and anatomical traits (Perez and Feeley, 2021; Ye et al., 2023). For example, plants can regulate leaf temperature by modifying leaf morphological and anatomical traits, such as increasing LMA and LT, or decreasing LA, which mitigates the damage caused by extreme temperatures and thereby influences heat tolerance (Slot et al., 2021; Zhou and Lin, 2023). Additionally, karst species often accumulate higher Ca and Mg in their leaves and display enhanced mechanical resistance (Fu et al., 2019; Wan et al., 2023, 2025), further supporting the role of lithological filtering in shaping PC1. Despite these clear patterns, a substantial portion of trait variation remained unexplained, which may be attributable to unaccounted environmental variables such as soil moisture and light intensity, trait plasticity.

### 4.3 Relationships among plant leaf physiological tolerance

Our findings show a positive correlation between heat and cold tolerance, indicating that species with greater heat tolerance also tend to withstand low-temperature stress. This contrasts with observations that plants from warmer regions often exhibit greater heat tolerance (Feeley et al., 2020; Slot et al., 2021). This relationship between thermal tolerance likely arises from shared physiological stresses, such as cellular structural damage, membrane dysfunction, and oxidative stress, imposed by both high and low temperature extremes, which drive plants to develop convergent adaptive strategies (Gill et al., 2010; Liu et al., 2017; Cheema and Garg, 2021). However, to our knowledge, no studies report a negative heat-cold tolerance relationship in woody plants, highlighting the need for further validation. We also found a positive correlation between cold and drought tolerance, potentially driven by seasonal water stress. Winter cold temperatures are often accompanied by reduced precipitation and soil moisture deficits, while summer droughts lead to an imbalance between transpiration demand and water supply, both of which challenge plants with insufficient water availability (Bray, 1997; Sato et al., 2024; Zhang et al., 2024a). This seasonal water stress drives plants to develop convergent adaptive strategies, such as reducing vessel diameter and enhancing xylem resistance to embolism (Lobo et al., 2018; Levionnois et al., 2021), thereby improving their survival in cold, arid environments. In contrast to Münchinger et al. (2023), we found no correlation between heat and drought tolerance. This decoupling may result from the differences in the timescales of plant responses to these stresses: heat waves typically last for short periods (hours to days), requiring rapid plant responses, such as inducing heat shock protein expression within hours to maintain photosynthetic and cellular structural stability (Havaux, 1993; Grossman, 2023), whereas drought persists over longer periods (weeks to months), compelling plants to gradually adapt to water deficits through mechanisms such as osmotic adjustment, increased water use efficiency, and root system modifications (Havaux, 1993; Grossman, 2023). These differences in rapid versus long-term responses may result in independent adaptive mechanisms for heat and drought tolerance, rendering the correlation between them insignificant. Overall, the interplay of synergistic co-tolerance and trade-offs among multiple physiological tolerances enables plants not only to adapt to a wide range of temperatures from heat to cold but also to cope with seasonal water stress, thereby expanding their ecological niches.

### 4.4 Associations between leaf physiological tolerance traits and morphological and anatomical traits

Both LT and LMA were positively correlated with heat tolerance (LT with T50_heat_ and Tcrit_heat_, LMA with T50_heat_), supporting the idea that greater structural investment enhances resistance to extreme heat (Zhang et al., 2024b). Thicker leaves may contain more cell layers and protective tissues or possess greater water storage capacity content to offset the effects of warming (Kröber et al., 2015; Leigh et al., 2017; Tserej and Feeley, 2021). Similarly, higher LMA typically indicates tougher tissues and longer leaf lifespan (Wright et al., 2004; Wang et al., 2023b), providing better mechanical support and structural stability, which enhances adaptability and enables plants to maintain normal photosynthesis under high-temperature stress (Sastry and Barua, 2017; Wan et al., 2023). These traits collectively enhance physiological tolerance, helping to mitigate damage caused by heat stress (Sastry et al., 2018; Zhang et al., 2024b). However, this study did not find a correlation between LA and heat tolerance, which is consistent with the conclusions of most scholars (Sastry and Barua, 2017; Wright et al., 2017; Ning et al., 2024), suggesting that the correlation between LA and heat tolerance is not universal and may be specific to certain high-temperature, arid environments (Leigh et al., 2017; Sastry and Barua, 2017).

Contrary to our initial expectations, LT, LA, and LMA showed no significant correlation with cold or drought tolerance. This may be because cold tolerance is often depends on physiological and biochemical adjustments (such as membrane stability, antifreeze proteins, and osmoprotectants) (Liu et al., 2017) and drought tolerance is more closely linked to water-use efficiency and hydraulic traits (Peters et al., 2021), whereas leaf morphological and anatomical traits, such as LT, LA, and LMA, vary primarily in response to local microhabitat conditions and selection pressures (Pérez et al., 2014; Li et al., 2022). Although earlier studies inferred associations between traits such as LT, LA, and LMA and drought tolerance based on π_tlp_, these associations were generally weak (Maréchaux et al., 2015; McGregor et al., 2021). Particularly in tropical rainforests, it is difficult to accurately infer plant drought tolerance solely from leaf morphology and anatomy (Maréchaux et al., 2020).

## 5 Conclusions

In summary, karst species exhibit greater drought tolerance but lower heat tolerance than non-karst species—a pattern driven primarily by lithological differences between habitats. In contrast, similar cold tolerance across both forest types appears to result from phylogenetic constraints. Both groups display synergistic adaptations to multiple stressors, with heat tolerance closely linked to leaf structural investment. These findings highlight the vulnerability of karst plants to increasing heat extremes under climate change, emphasizing the urgent need to prioritize the conservation of these unique habitats, especially given their high endemic species richness. Future work should focus on elucidating the interplay of multiple stress factors and trait-based synergies, delving into the physiological and molecular mechanisms underlying their stress responses, thereby improving our ability to predict their persistence under future climate change.

## CRediT authorship contribution statement

**Qiufeng Ning**: Conceptualization, Methodology, Formal analysis, Investigation, Writing— Original Draft. **Hui Liu**: Writing—Review & Editing. **Jiawei LI**: Writing—Review & Editing. **Yunpeng Nie**: Resources, Funding acquisition. **Yin Wen**: Conceptualization, Methodology, Resources, Writing—Review & Editing, Funding acquisition.

## Declaration of competing interest

The authors declare that they have no known competing financial interests or personal relationships that could have influenced the work reported in this paper. All authors have approved the submission of this manuscript to Plant Diversity.
