# Supplementary material for: Differences in leaf heat and drought tolerance but not cold tolerance between karst and non-karst forest plants
Source: Plant Divers. 2025 Aug 26;48(2):363–72. doi: 10.1016/j.pld.2025.08.006 (PMC13071437; doi:10.1016/j.pld.2025.08.006)
Supplement: Multimedia component 1 [file mmc1.doc]

**Supplementary materials**

**Table S1:** **Coordinates and climatic data for sampling sites in two habitats.** Mean annual temperature (MAT), maximum temperature of the warmest month (TmaxWM), minimum temperature of the coldest month (TminCM), mean annual precipitation (MAP), precipitation of the wettest quarter (PWQ), and precipitation of the driest quarter (PDQ) were extracted from CHELSA V.2.1 (Br*un et a*l. 2022a, Br*un et a*l. 2022b).

| **Habitat** | **Location** | **Elevation**  **(m)** | **MAT**  **(**°C**)** | **TmaxWM**  **(**°C**)** | **TminCM**  **(**°C**)** | **MAP**  **(mm)** | **PWQ**  **(mm)** | **PDQ**  **(mm)** |
| --- | --- | --- | --- | --- | --- | --- | --- | --- |
| Karst | 22.47^o^N,  106.95^o^E | 273 | 21.8 | 30.2 | 9.7 | 1666.2 | 871.4 | 120.2 |
| Non-karst | 21.84^o^N,  107.89^o^E | 558 | 19.3 | 26.8 | 8.7 | 1946.6 | 1764.7 | 152.0 |

**Table S2:** **Relative contributions of physiological tolerance traits, principal components PC1 and PC2 in karst and non-karst species based on lithology and phylogeny.** Abbreviations are in Table 1.

| **Trait** | **Lithology** | **Interaction**  **effects** | **Phylogeny** | **Unexplained**  **variance** |
| --- | --- | --- | --- | --- |
| Tcrit_heat_ | 0.287 | 0.181 | 0.052 | 0.480 |
| T50_heat_ | 0.150 | -0.046 | 0.132 | 0.763 |
| Tcrit_cold_ | 0.128 | -0.029 | 0.154 | 0.747 |
| T50_cold_ | 0.126 | -0.032 | 0.188 | 0.717 |
| π_tlp_ | 0.279 | 0.107 | 0.010 | 0.604 |
| PC1 | 0.208 | 0.201 | 0.106 | 0.486 |
| PC2 | 0.100 | -0.043 | 0.183 | 0.760 |


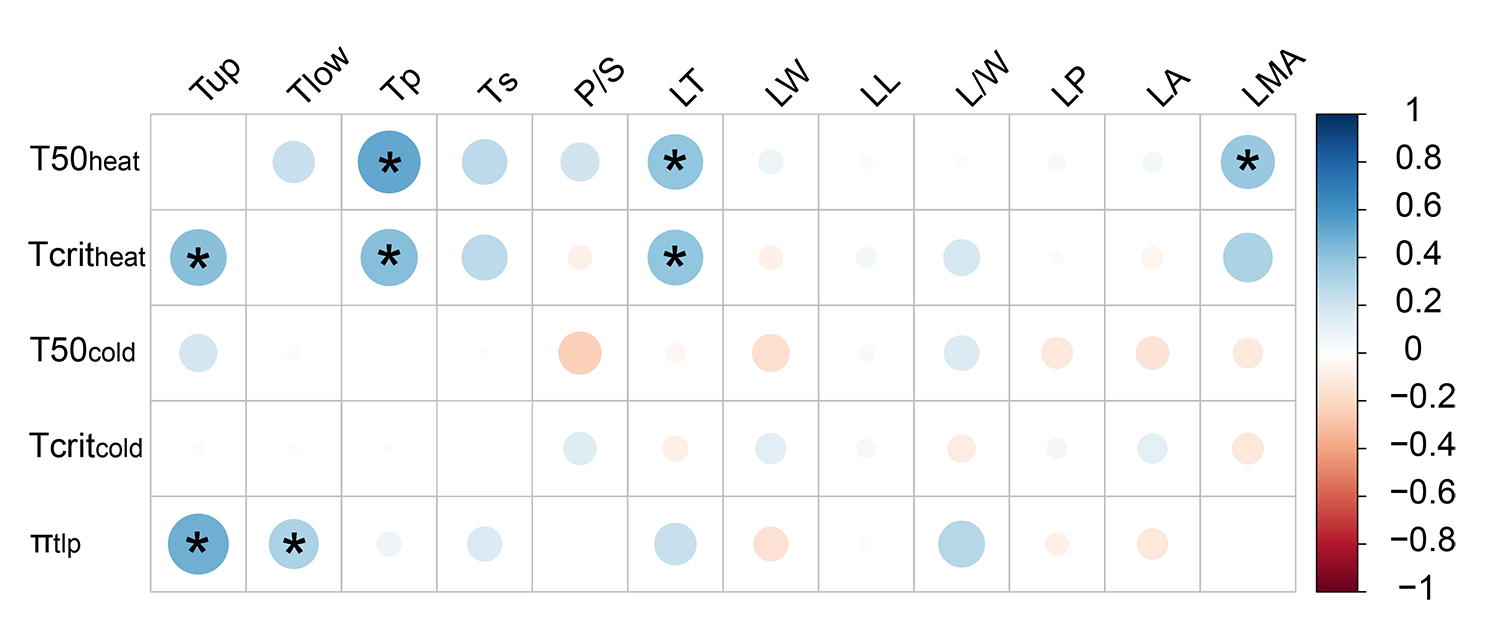


**Figure S1:** **Pearson correlations of leaf morphological traits with different tolerance indicators in 39 evergreen woody species of Karst and Non-karst.** *, p < 0.05. Abbreviations are in Table 1.


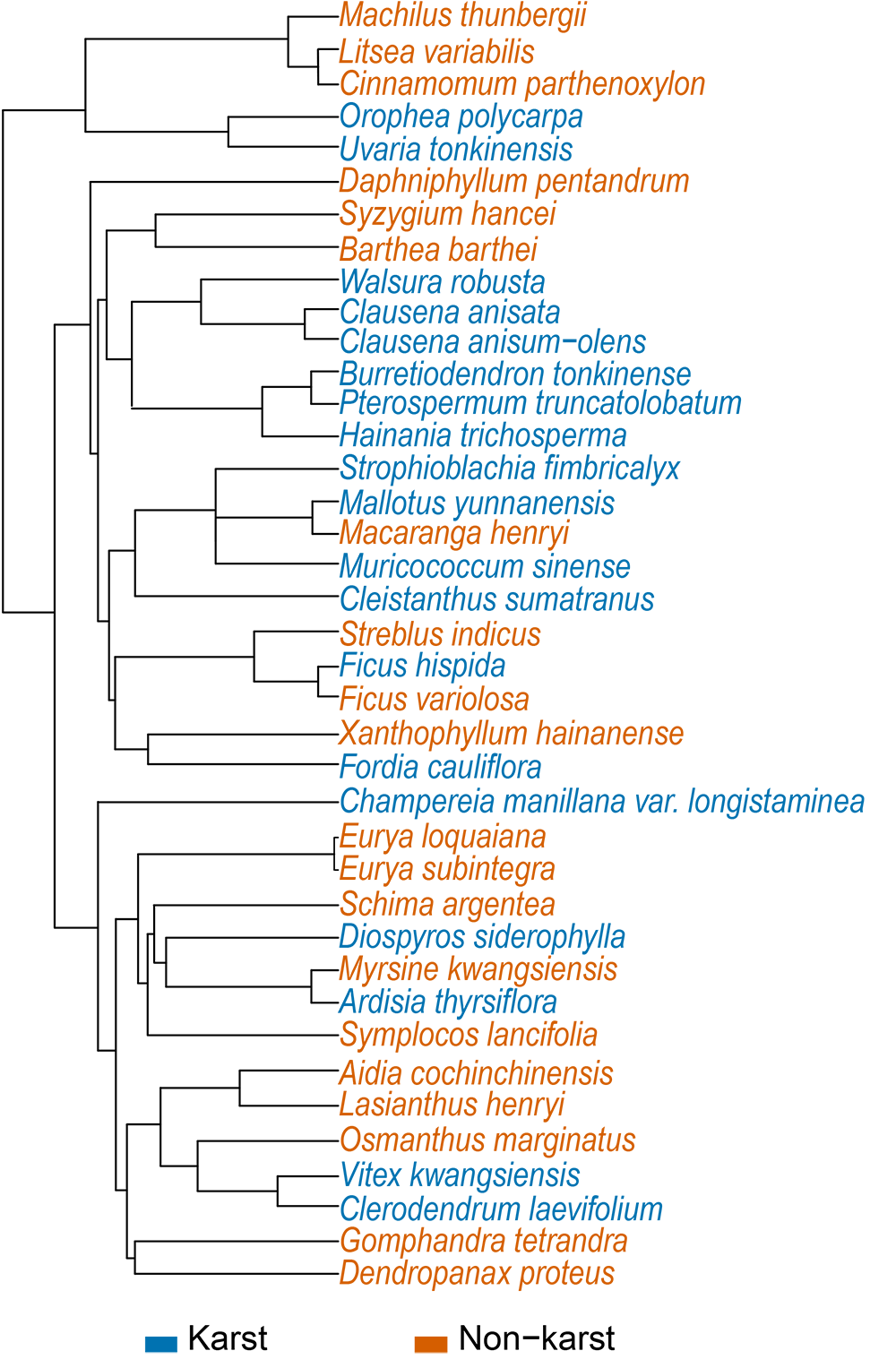


**Figure S2:** **Phylogenetic tree of 19 karst (blue) and 20 non-karst (orange) plant species.**
